# *Waddlia chondrophila* induces systemic infection, organ pathology, and elicits Th1-associated humoral immunity in a murine model of genital infection

**DOI:** 10.3389/fcimb.2015.00076

**Published:** 2015-11-04

**Authors:** Sam Vasilevsky, Joel Gyger, Alessandra Piersigilli, Ludovic Pilloux, Gilbert Greub, Milos Stojanov, David Baud

**Affiliations:** ^1^Materno-fetal and Obstetrics Research Unit, Department of Obstetrics and Gynecology, Maternity, University HospitalLausanne, Switzerland; ^2^School of Life Sciences, École Polytechnique Fédérale LausanneLausanne, Switzerland; ^3^Institute of Animal Pathology, University of BernBern, Switzerland; ^4^Centre Hospitalier Universitaire Vaudois, Institute of Microbiology, University HospitalLausanne, Switzerland

**Keywords:** *Chlamydia*-like, adverse pregnancy outcome, genital tract infection, intracellular bacteria, zoonosis

## Abstract

*Waddlia chondrophila* is a known bovine abortigenic *Chlamydia*-related bacterium that has been associated with adverse pregnancy outcomes in human. However, there is a lack of knowledge regarding how *W. chondrophila* infection spreads, its ability to elicit an immune response and induce pathology. A murine model of genital infection was developed to investigate the pathogenicity and immune response associated with a *W. chondrophila* infection. Genital inoculation of the bacterial agent resulted in a dose-dependent infection that spread to lumbar lymph nodes and successively to spleen and liver. Bacterial-induced pathology peaked on day 14, characterized by leukocyte infiltration (uterine horn, liver, and spleen), necrosis (liver) and extramedullary hematopoiesis (spleen). Immunohistochemistry demonstrated the presence of a large number of *W. chondrophila* in the spleen on day 14. Robust IgG titers were detected by day 14 and remained high until day 52. IgG isotypes consisted of high IgG2a, moderate IgG3 and no detectable IgG1, indicating a Th1-associated immune response. This study provides the first evidence that *W. chondrophila* genital infection is capable of inducing a systemic infection that spreads to major organs, induces uterus, spleen, and liver pathology and elicits a Th1-skewed humoral response. This new animal model will help our understanding of the mechanisms related to intracellular bacteria-induced miscarriages, the most frequent complication of pregnancy that affects one in four women.

## Introduction

Approximately 25% of women will experience a miscarriage in their lives, representing the most frequent complication in early pregnancies. Although the arrays of diagnostic tests and possible therapeutic interventions in the management of miscarriage have grown significantly, a cause is identified in only 50% of the cases (Carrington et al., 2005; Baud and Greub, 2011). Genetic factors are the most frequent causes, being identified in about 25% of cases (Regan and Rai, 2000; Rai and Regan, 2006). The proportion of miscarriages attributed to infection is approximately 10–15% (Penta et al., 2003; Baud et al., 2008), but this proportion might be underestimated. Intracellular pathogens, which are not detected on routine culture media, might thus represent possible agents of miscarriages with unknown etiology (Baud and Greub, 2011). *Chlamydia trachomatis* infections have been associated with miscarriages, ectopic pregnancies, and neonatal pneumonia (Vincent et al., 1998; Baud and Greub, 2011). Likewise, *Waddlia chondrophila* has been recently associated with adverse pregnancy outcomes both in humans and animals (Baud et al., 2007, 2008, 2011, 2014; Hornung et al., 2015). *W. chondrophila* belongs to the Chlamydiales order (Rurangirwa et al., 1999; de Barsy and Greub, 2013) and was originally identified in aborted bovine fetuses from the United States (Dilbeck et al., 1990) and Germany (Henning et al., 2002). Similarly to *C. trachomatis, W. chondrophila* is an obligate intracellular bacterium and exhibits a biphasic lifecycle (de Barsy and Greub, 2013). *In vitro* studies have demonstrated the ability of *W. chondrophila* to infect a variety of cell lines, including endometrial cells and primary human macrophages (Goy et al., 2008; Croxatto and Greub, 2010; Kebbi-Beghdadi et al., 2011a,b), supporting its role in miscarriage. Moreover, recent studies reported a higher seroprevalence of anti-*W. chondrophila* immunoglobulin G found in patients with miscarriage compared to control groups (Baud et al., 2007, 2014).

Several animal models were developed for the study of genital infection of bacteria belonging to the *Chlamydia* genus, thus helping the comprehension of pathologies caused by these bacteria (Vasilevsky et al., 2014). At the contrary, the lack of experimental data and animal models of infection limit our understanding of the disease caused by *W. chondrophila*. For this reason, we developed a female mouse model to investigate the nature of *W. chondrophila* genital infection in order to study the pathology and the immune system response induced by this emerging human pathogen.

## Materials and methods

### *W. chondrophila* culture

*W. chondrophila* was cultured and purified as previously described (Goy and Greub, 2009). Briefly, *W. chondrophila* strain ATCC VR-1470 was cultured in *Acanthamoeba castellanii* grown in 75 cm^2^-cell culture flasks (Corning, NY, USA) at 32°C in Peptone–Yeast–Glucose (PYG) broth as previously described (Greub and Raoult, 2002). Cell cultures were then harvested and filtered through a 5-μm filter (Merck Millipore, Darmstadt, Germany) to eliminate the remaining amoebae.

### Infection procedure

All animal experiments were approved by the “Office Vétérinaire du Canton de Vaud,” Lausanne, Switzerland (authorizations n° 2090.0 and 2090.1) and performed according to our institutional guidelines for animal experiments.

Female C57BL/6 mice (8 weeks old) were obtained from Charles River Laboratories (L'Arbresle, France) and housed under pathogen-free conditions at the animal facility of the CHUV (Centre Hospitalier Universitaire Vaudois, Lausanne, Switzerland). Before infection, mice were treated subcutaneously with 0.1 μg estradiol (Sigma-Aldrich Chemie, Buchs, Switzerland) and 2 mg medroxyprogesterone acetate (Depo-Provera, Pfizer, New York, USA). One week later, mice were anesthetized with rompun (Bayer, Leverkusen, Germany) and ketasol (Dr. E. Graeub AG, Bern, Switzerland) and injected with live 10^9^, 10^7^, 10^5^, or 10^9^ heat-inactivated (HI) *W. chondrophila* into the uterine horns using a semi-rigid cannula (Introcan, B Braun, Melsungen, Germany; Supplementary Figure 1B). Heat inactivation was performed by placing bacteria in a 95°C heat block for 1 h. A mock group containing filtered PYG from disrupted amoebae culture was included as a negative control.

For qPCR analysis, two individual experiments with *n* = 3 and 5 (total *n* = 8) for each condition, including a mock control (*n* = 4), were performed. For microimmunofluorescence analysis, two individual experiments with five infected mice were analyzed for each condition (total *n* = 10) and 9 mock controls (*n* = 9). Evaluation of pathological outcomes and severity scores of *W. chondrophila* infection were done on four mice (*n* = 4) and 4 mock controls (*n* = 4). Time course experiments for the assessment of IgG response and determination of IgG isotypes, included a total of 12 infected mice (*n* = 12) and 12 mock controls (*n* = 12).

### Organ collection

Organs were collected at days 7, 14, and 21 post-infection to measure bacterial burden and subsequently frozen at −80°C. For liver, spleen and muscle, a small section (~2 mm^2^) was used for DNA extraction and qPCR. The entire lymph node was used for qPCR. Organ samples used for McCoy cell co-culture were utilized immediately after harvest (i.e., not frozen).

### Quantitative RT-PCR

DNA was extracted using the Wizard SV Genomic DNA purification system (Promega, Wisconsin, USA). All samples were tested with a 16S rRNA *W. chondrophila* specific real-time qPCR as previously described (Goy et al., 2009).

### Microimmunofluorescence

Blood samples were collected from mice anesthetized by isofluorane (Attane, Isofluorane ad us. vet., Piramal Healthcare, India). Blood samples were collected in Microvette 300 LH tubes (Sarstedt, Nümbrecht, Germany). After centrifugation for 5 min at 2000 × g, plasma (supernatant) was collected in a separate tube and stored at −20°C. Microimmunofluorescence (MIF) was used to detect serum anti-*Waddlia* antibodies using formalin inactivated (0.3%) *W. chondrophila* strain ATCC VR-1470 as antigen. Serum samples were diluted 1:8, 1:16, 1:32, 1:64, 1:256, and 1:512 using Phosphate-buffered saline (PBS) and 1% Bovine serum albumin (BSA, Serva, Heildeberg, Germany). Goat anti-mouse IgG, IgG2a, IgG3, and IgG1 secondary antibodies conjugated to Alexa Fluor 488 dye (Molecular Probes/Life Technologies, USA, diluted 1/1000) were used to detect the presence of IgG, and IgG isotypes.

### Immunohistochemistry

Tissue samples or whole organs were fixed in 4% buffered paraformaldehyde (Paraformaldehyde, Electron Microscopy Sciences, PA, USA) and embedded in paraffin. Embedded samples were cut in 5-μm-thick sections on a microtome. Paraffin was removed by two xylene (Sigma-Aldrich Chemie) washes and samples were rehydrated using a graded ethanol series (100, 95, 80, and 70%) and finally rehydrated in PBS. Rehydrated sections were blocked using PBS supplemented with 10% goat normal serum (Interchim, Montluçon, France), 5% BSA and 0.3% triton X-100 (Applichem, Darmstadt, Germany) for 1-h at room temperature (RT) in a wet chamber. After three washes for 5 min each in PBS, 50 μl of blocking solution supplemented with rabbit anti-*W. chondrophila* polyclonal antibody (Kebbi-Beghdadi et al., 2011a, diluted 1/1000) was added to the slides in a wet chamber and incubated for 1-h at RT. Negative control was performed with the pre-immunization serum from the rabbit used to produce the anti-*W. chondrophila* polyclonal antibody. After three washing steps for 5 min each in PBS, the secondary antibody (goat anti-rabbit Alexa Fluor 488-conjugated, diluted at 1/1000 in blocking solution, Molecular Probes/Life Technologies), and DAPI (4′,6-diamidino-2-phenylindole, diluted at 1/1000 in blocking solution, Molecular Probes/Life Technologies) were added and incubated for 1-h at RT in a wet chamber protected from light. After three final washes with PBS and one with deionized water, slides were mounted using Mowiol 4-88 (Sigma-Aldrich Chemie), dried overnight and subsequently used for microscopy analysis.

### Epifluorescence microscopy

Epifluorescence microscopy was performed using an Axiovision epifluorescence microscope (Zeiss, Jena, Germany).

### Assessment of *W. chondrophila* viability from organs in mccoy cells

Spleen and liver samples (~2 mm^2^) were collected and cell suspensions were made by mechanical disruption and passage through a 70 μm nylon cell strainer (Falcon Corning, New York, USA) with 2 ml Dulbecco's Modified Eagle Medium supplemented with sodium pyruvate and glutamine (GE Healthcare, Glattbrugg, Zurich, Switzerland) and 10% Fetal bovine serum (FBS; Connectorate AG, Dietikon, Switzerland). Cell suspensions were transferred to a 50 ml conical tube containing glass beads and vortexed to crack open cells. 100 μl of cell suspension were transferred to a 24-well plate containing McCoy cells at confluence in DMEM supplemented medium, immediately centrifuged at 1790 RCF for 10 min and placed in an incubator (37°C, 5% CO_2_) for 3 h. After incubation, 50 μg/ml of gentamycin (Bioconcept, Allschwil, Switzerland) and 30 μg/ml of ampicillin (Ampicillin sodium salt, Sigma-Aldrich Chemie, Buchs, Switzerland) were added to the samples in order to inhibit growth of extracellular and other intracellular bacteria, respectively, as *W. chondrophila* is resistant to ampicillin at >32 μg/ml due to the presence of a β-lactamase (Goy and Greub, 2009; Bertelli et al., 2010). Viability was assessed by qPCR and by light microscopy examination of McCoy cells 3 h or 7 days post-inoculation.

### Histopathological analysis

Uterine horns, liver, and spleen from mock and infected mice were removed at indicated time points postinfection (p.i.) and fixed in 4% buffered formalin and embedded in paraffin for histopathological analysis. The tissue sections were stained with Hematoxylin & Eosin (H&E) at the Mouse Pathology Facility (University of Lausanne, Switzerland) and sent for morphologic evaluation, in a blind fashion, to a board certified veterinary pathologist (P.A.). Pathologic changes were scored according to severity on a numerical scale ranging from 0 to 4, where 0 indicates absence of lesions and 4 the highest grade.

### Statistical analysis

Data were analyzed using the GraphPad Prism 6 software (GraphPad Software Inc., San Diego USA). When necessary, groups were compared using the Mann–Whitney rank sum test. Differences with a *P*-value of < 0.05 were considered statistically significant. The level of significance is depicted as ^*^P < 0.05; ^**^P < 0.01. Data are presented as means ± standard error of mean (SEM).

## Results

### *W. chondrophila* infectious dose and heat-inactivation

**Figure 1:**
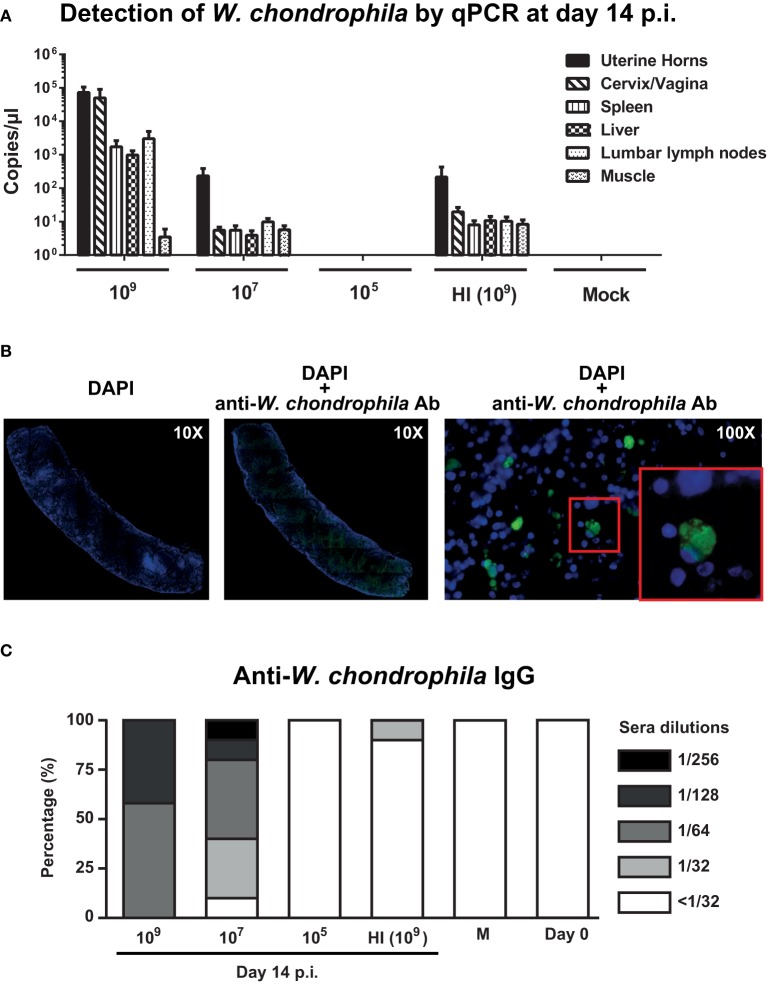
**Bacterial burden in genital and non-genital organs and anti-***W. chondrophila*** IgG titers**. Mice were infected with decreasing amounts of live (10^9^, 10^7^, 10^5^) and HI (10^9^) *W. chondrophila*. **(A)** Organ bacterial burden measured by qPCR on day 14 p.i. qPCR values represent the mean SEM. Results are representative of two independent experiments with *n* = 3 and 5 for each experiment. **(B)** Detection of *W. chondrophila* by immunofluorescence in a spleen section at day 14 p.i. Left: visualization of mouse cells by DAPI (10X). Middle: co-visualization of mouse cells by DAPI and bacteria by a specific anti-*W. chondrophila* antibody (10X). Right: magnification of the co-visualization experiment (100X). Red square depicts a 200X magnification. **(C)** IgG measured by MIF at day 14 p.i. Day 0 represents naive mice before challenge with *W. chondrophila* and M represents mock challenged mice. Results are representative of two individual experiments with *n* = 5 for each experiment.

We first investigated whether a dose response or heat inactivation (HI) modulate pathogenicity of *W. chondrophila* and humoral immunity in a mouse genital infection model. Mice were inoculated via the uterine horns with HI bacteria (10^9^) and decreasing amounts (10^9^, 10^7^, 10^5^) of live bacteria and sacrificed on day 14 postinfection (p.i.; Figure 1 and Supplementary Figure 1). Bacterial load in organs was then assessed by specific *W. chondrophila* qPCR. Infection with live *W. chondrophila* resulted in high bacterial loads in genital but also in non-genital organs including spleen, lumbar lymph nodes and liver. Decrease of the inoculum from 10^9^ to 10^7^ resulted in a bacterial load reduction of >9000-fold in cervix/vagina and >300-fold in the uterine horns and non-genital organs (Figure 1A). Furthermore, bacterial burden in mice infected with 10^9^ HI *W. chondrophila* was also severely reduced in all organs compared to mice infected with 10^9^ live bacteria (Figure 1A). Surprisingly, we were not able to detect any bacteria in organ samples from mice that were infected with 10^5^ live *W. chondrophila* (Figure 1A).

Presence of *W. chondrophila* in organ sections of mice infected with 10^9^ bacteria was confirmed using immunohistochemistry. Figure 1B depicts a spleen section in which bacteria were detected using a *W. chondrophila* specific antibody 14 days post-infection.

Analysis of the IgG response, showed strong anti-*Waddlia* titers in mice genitally challenged with 10^9^ and 10^7^ live bacteria (Figure 1C). Mean humoral response of mice challenged with HI bacteria was below the cut-off dilution of 1:32 and a total abrogation of anti-*Waddlia* response was observed for mice infected with 10^5^ live bacteria (Figure 1C and Supplementary Table 1). Because of the high bacterial burden and robust titers from 10^9^-infected mice, we used this dose for all subsequent experiments in this study.

### *W. chondrophila* genital infection results in high bacterial burden in genital and non-genital organs

**Figure 2:**
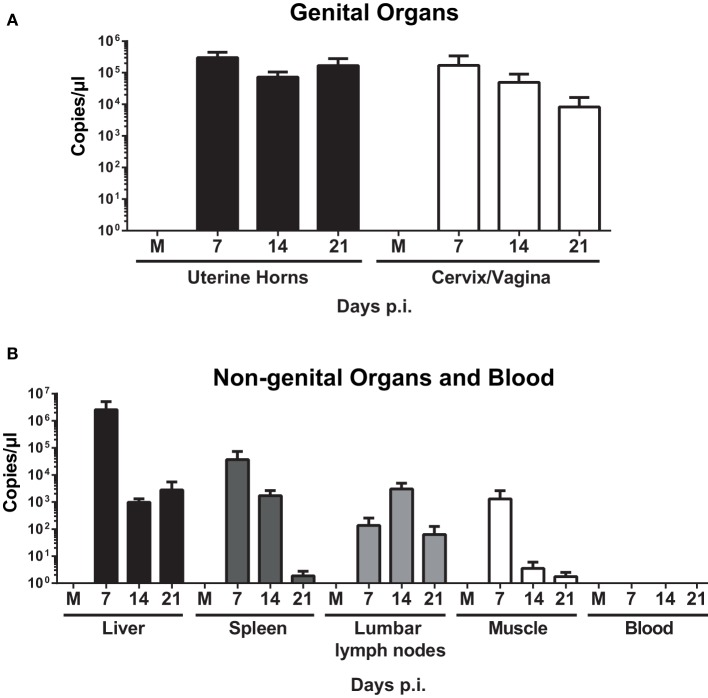
**Quantification of ***W. chondrophila*** in genital and non-genital organs**. Mice were infected with live 10^9^ *W. chondrophila* or mock (M). **(A)** Bacterial burden in uterine horns and cervix/vagina at days 7, 14, and 21 p.i. **(B)** Bacterial burden in liver, spleen, lumbar lymph nodes, muscle, and blood at days 7, 14, and 21 p.i. qPCR values represent the mean SEM. Results are representative of two independent experiments with *n* = 3 and 5 for each experiment.

qPCR analysis on uterine horns and cervix/vagina samples demonstrated a high bacterial burden on days 7, 14, and 21 p.i. (Figure 2A). Infection also spread to lumbar lymph nodes, spleen, and liver (Figure 2B), but bacteria were never identified in blood (data not shown). To assess whether the bacteria in those organs were able to replicate, spleen, and liver homogenates were incubated with McCoy cells for 7 days, but no replication was observed (data not shown). Vaginal shedding was resolved 7 days post-infection (< 10^1^ copies/μl), and replicating bacteria were still present at days 2 and 4 post-infection (data not shown).

### Organ pathology in *W. chondrophila* infected mice

Mice genitally infected with 10^9^ *W. chondrophila* exhibited not only splenomegaly and hepatomegaly, but also lymphadenopathy and severe metritis (Supplementary Figure 2). Day 14 coincided with the highest percentage of mice that exhibited splenomegaly (50%), lymphadenopathy (87%), and hepatomegaly (50%). However, day 21 had the highest percentage of mice that demonstrated salpingitis (25%). Mock mice and mice infected with 10^9^ HI *W. chondrophila* did not demonstrate overt organ pathologic changes.

**Figure 3:**
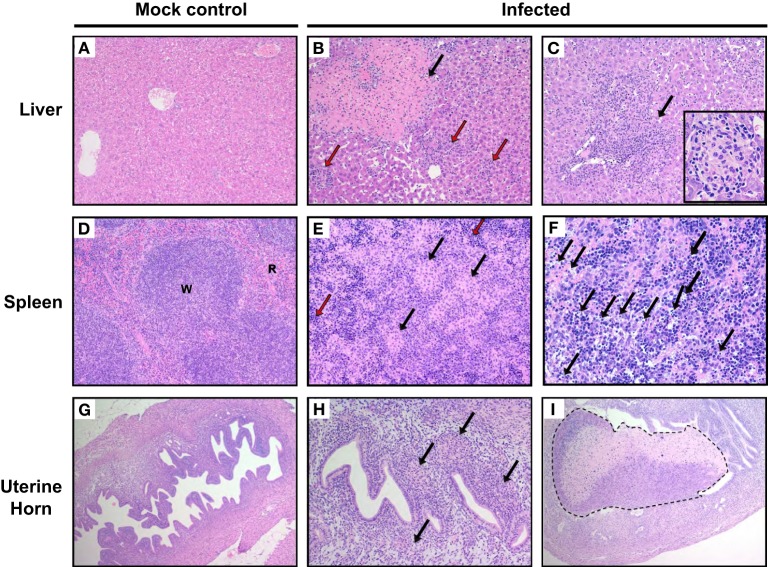
**Pathological outcomes in mice infected with ***W. chondrophila*****. Liver, spleen, and uterine horn tissues of mice infected with 10^9^ *W. chondrophila* and mock were collected at day 14 p.i. and analyzed for histological changes by H&E staining under light microscope. **(A)** Liver (200X): normal hepatic parenchyma in a control mouse. **(B)** Liver (200X): a large area of perivascular parenchymal coagulative necrosis (arrow) and leukocytic infiltration (red arrows) is observed in an infected mouse 14 days after infection. **(C)** Liver (200X): perivascular coalescing foci of histiocytic and neutrophilic aggregates within the hepatic parenchyma (arrow). The insert depicts a granuloma (400X). **(D)** Spleen (200X): normal distribution of white (W) and red pulp (R) in a control animal. **(E)** Spleen (200X): the white and red pulp are diffusely replaced by nodular aggregates of histiocytes (arrow) in a infected mouse. A prominent EMH response is visible in the remnants of red pulp (red arrow). **(F)** Spleen (400X): increased apoptotic cells in the red and white pulp of an infected mouse (arrows). **(G)** Uterus (100X): normal appearance of the uterus in a control mouse. **(H)** Uterus (200X): multifocal and periglandular infiltration of histiocytes (arrow) and few neutrophils in the endometrium of a *W. chondrophila* infected mouse. **(I)** (100X): left uterine horn of an infected mouse: a large deciduoma undergoing degeneration obliterates almost completely the lumen of the organ (dashed line). Results are representative of four individual mice.

To characterize microscopic lesions induced by *W. chondrophila* infection, liver, spleen and uterine horns from mice 14 days p.i. from both 10^9^ infected and mock challenged mice were analyzed (Figures 3A–I). Liver from infected mice exhibited leukocytes infiltration: mostly neutrophils and macrophages (the latter increasing over time) coalescing into histiocytic nodules or granulomas (Figures 3B,C) and variable sized areas of coagulative hepatocellular necrosis. 75% of mice exhibited macrophage infiltration on day 7, 50% on day 14, and 75% on day 21 in the liver (Supplementary Table 2). In spleens of infected mice, both the white and red pulps were replaced by multifocal histiocytic nodules (Figure 3E, arrows). Extramedullary hematopoiesis (i.e., hematopoiesis that occurs in organs other than bone marrow, EMH) was quite prominent as shown in Figure 3E (red arrows). Increased number of apoptotic cells was observed in the red and white pulp (Figure 3F). Histiocytes and neutrophils were present in the endometrium of infected mice (Figure 3H arrows), but often they were found also within the myometrium and the serosa. Necrotic deciduomas were observed in the lumen of infected animals only (Figure 3I).

**Figure 4:**
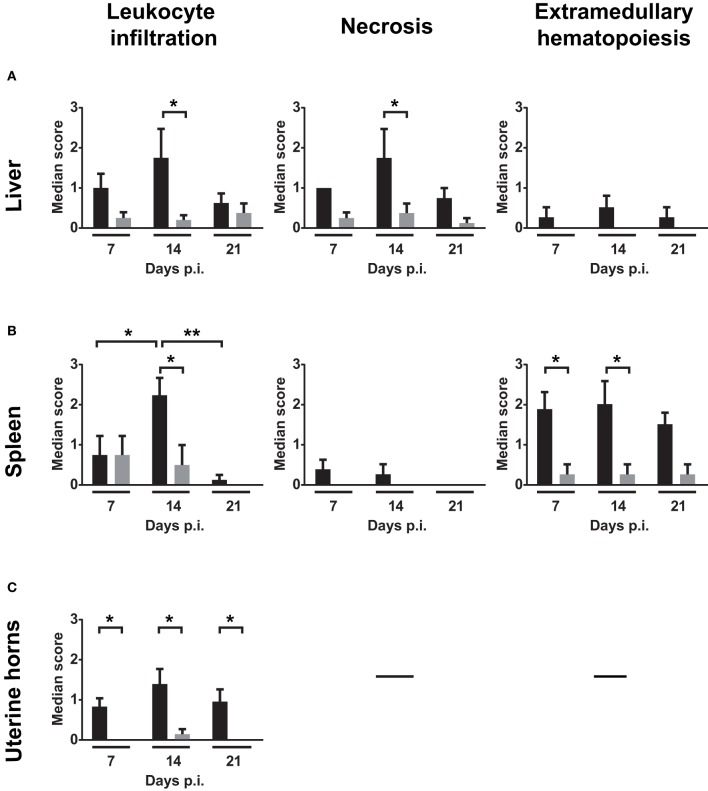
**Median severity scores of spleen, liver and uterine horn pathology and leukocyte infiltration**. Liver, spleen, and uterine horn tissues of infected mice and mock were collected at days 7, 14, and 21 p.i. and analyzed for histopathological changes by H&E staining under light microscope. Severity scores (1–4) of inflammatory cell infiltrates, necrosis and EMH are shown for **(A)** liver, **(B)** spleen, and **(C)** uterine horns. Scores are representative of four individual mice. Groups were compared using the Mann–Whitney rank sum test. The level of significance is depicted as ^*^P < 0.05; ^**^P < 0.01.

Liver, spleen, and uterine horn pathology severity scoring for necrosis, leukocyte infiltration and EMH were assessed on days 7, 14, and 21 p.i. (Figure 4). Figures 4A,B show that necrosis is more severe in the liver than in the spleen at days 7, 14, and 21 p.i. On day 14, incidence of liver changes such as necrosis and neutrophilic infiltration was 100% in infected mice (Supplementary Table 2). Severity median scores (Figure 4) demonstrated the trend of the *W. chondrophila*-associated lesions in infected mice reaching a peak on day 14 and decreasing on day 21. A significant difference was also seen when compared to mock-infected mice (*p* < 0.005 at day 14 for leukocyte infiltration in all organs, liver necrosis and spleen EMH).

### *W. chondrophila* genital infection elicits a robust IgG and predominately Th1-associated antibody response

**Figure 5:**
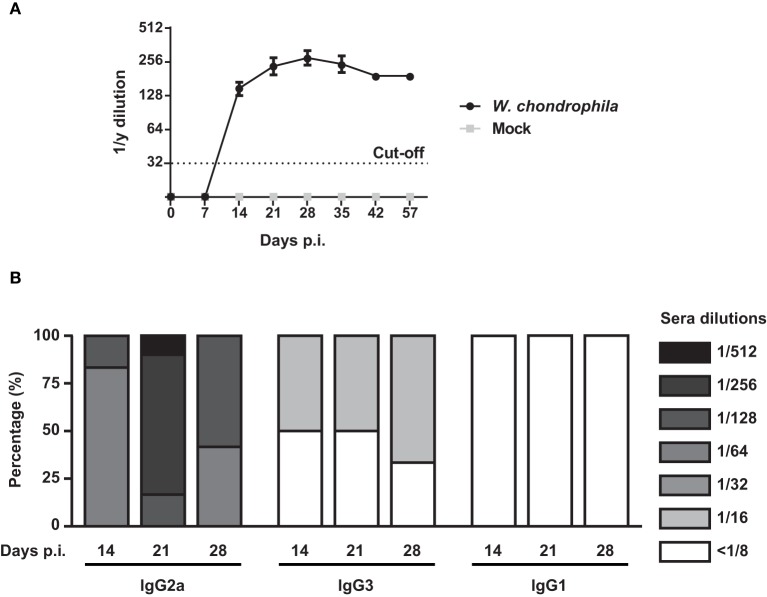
**IgG response and detection of IgG isotpyes from sera in ***W. chondrophila*** infected mice**. Mice were infected with 10^9^ *W. chondrophila* or mock. **(A)** IgG titers from sera were measured by MIF on days 0, 7, 14, 21, 28, 35, 43, 57 p.i. **(B)** Comparison of MIF IgG2a, IgG3, and IgG1 from infected mice on days 7, 14, 21, 28, 35 p.i. Data are representative of 12 individual mice.

Analysis of anti-*W. chondrophila* antibody response demonstrated that specific IgG antibodies were present by day 14, peaked on day 28, and slightly declined by day 42 (Figure 5A and Supplementary Table 2). Furthermore, elevated titers were present for at least 6 weeks. Mice challenged with mock did not elicit anti-*Waddlia* specific antibodies. Figure 5B demonstrates that the IgG response elicited by *W. chondrophila* was mainly comprised of the IgG2a isotype and to a lesser extent of the IgG3 isotype, indicating a Th1-associated humoral response.

## Discussion

*W. chondrophila* is an intracellular *Chlamydia*-related bacterium that has been strongly implicated in adverse pregnancy outcomes in women (Baud et al., 2007, 2011, 2014; Hornung et al., 2015). However, little is known how genital *W. chondrophila* infection spreads, elicits an immune response, and induces pathology. *Chlamydia* mouse infection models have demonstrated that *Chlamydia muridarum* is a natural pathogen of mice whereas *C. trachomatis* is only infectious in mice if infected in high doses directly in the uterine horn (reviewed in Vasilevsky et al., 2014). Since, this is the first reported instance of a *W. chondrophila* genital mouse infection model and since *W. chondrophila* is closely related to *C. trachomatis*, we used a semi-rigid cannula per vaginam to pass the cervix and injected live freshly cultured *W. chondrophila* into the uterine horn to increase the chances of a progressive genital infection.

We first wanted to establish the optimal dose for a progressive bacterial infection and investigate whether decreasing amounts of live or HI *W. chondrophila* modulated the bacterial load in the organs and the antibody production. We determined that 10^9^ live *W. chondrophila* was the optimal dose for a genital infection, due to the induction of a pathological state and the generation of a robust antibody response. Lack of IgG titers in mice infected with 10^5^ bacteria might be explained by the fact that this infectious dose may not recruit enough dendritic cells (DCs) to initiate an IgG specific humoral response. Indeed, this hypothesis is supported by a study conducted by Maxion et al. where a decrease of *C. muridarum* infection resulted in a decrease of CD11c^+^ DC recruitment (Maxion et al., 2004).

Heat inactivation often denatures protein epitopes, which may affect protein binding onto MHC class I/II on T cells and subsequent production of important Th1-associated cytokines (Yu et al., 2011; Ding et al., 2014). Since, MHC class II binding of proteins on antigen presenting cells and subsequent presentation to T cells is critical for initiating a Th1 humoral response, this may be a plausible explanation for the low IgG titers in mice that were immunized with HI *W. chondrophila*. Mice challenged with *W. chondrophila* were used to investigate the bacterial burden in uterine horns, lumbar lymph nodes, spleen, and liver over a 3-week time period. High bacterial loads were observed throughout the experiment in the organs. The main limitation of the qPCR analysis used in our experiments is the fact that this technique detects *W. chondrophila* DNA, thus indirectly representing bacteria. Therefore, our data might indicate that bacterial DNA is present in the organs but not as intact EBs outside cells or RBs inside cells. In addition, we might also be measuring bacterial DNA that is present inside immune cells such as neutrophils, macrophages, or DCs. Spleen and liver homogenates were incubated with McCoy cells in order to assess whether these tissues contained *W. chondrophila* that were able to replicate. However, the clear evidence of bacteria by immunohistochemistry (day 14 p.i.) and the absence of replication in McCoy cells suggest that *W. chondrophila* remains in a non-replicating persistent form. The same has been observed for aberrant bodies of *C. trachomatis* when T cell-mediated immune response is induced and interferon (IFN-γ) is secreted (Baud et al., 2008). Since we demonstrated a high Th1-mediated immune response, IFN-γ levels might delay or abort differentiation into replicative (RB) and infectious (EB) stages.

None of the mock or infected mice lost weight or showed any overt signs of distress, highlighting the asymptomatic nature of a *W. chondrophila* genital infection. However, a high percentage of mice exhibited macroscopic lesions with an overall highest frequency at day 14l. Mock challenged mice did not show any evidence of gross changes at necropsy, including absence of granulomas in examined organs. The multiorgan involvement in infected mice at both macro- and histopathologic level points toward a dissemination from the uterus to tissues. The predominance of neutrophils followed by macrophages in the inflammatory infiltrate is consistent with the bacterial origin of the insult (Kim et al., 2008). Moreover, formation of granulomas has been previously associated with the presence of intracellular pathogens (Pettengill et al., 2009). The shift of the predominant leukocytic cell type over time correlates with the kinetics of the infection and inflammatory process, which evolves from a subacute (7 days) to a more chronic stage. Necrotic deciduomas were observed in the lumen of infected animals only, but the significance of such lesion is not clear. Occurrence of deciduomas is described in the literature in rodents as hyatrogenic effect of inoculation or intrauterine luminal delivery of substances or trauma/luminal manipulations in pseudopregnant animals (Finn and Keen, 1963; Hirabayashi et al., 1999). Another common cause is administration of sexual hormones for estrous cycle manipulation such as pseudopregnancy induction. As mock mice did receive the same manipulations and treatments, except for infection with *W. chondrophila*, a pathogenic correlation between the lesion and the etiologic agent cannot be excluded. In the present study all deciduoma were necrotic and undergoing degeneration. Apoptosis and regression is reported as normal event at about 10 days in literature (Hirabayashi et al., 1999). However, a direct effect of *W. chondrophila* infection can be hypothesized as similar bacteria, such as *C. trachomatis*, can infect decidual cells and lead to adverse pregnancy outcome (de la Torre et al., 2009). Validation of this hypothesis and dismissal of spontaneous degeneration of the decidual reaction would need further investigation.

A granulomatous inflammation is a known response to infection caused by intracellular bacterial organisms (Pettengill et al., 2009). The nodular arrangement of histiocytes mirrors the gross raised gray-white foci in the liver and in the spleen and is characterized by a Th-1 type immune response, which in the present study is further corroborated by the immunological data in our study.

Mice genitally infected with live *W. chondrophila* elicited a robust long-lasting systemic IgG humoral response that consisted mainly of IgG2a and IgG3 isotypes, which is characterized as Th1-associated response in mice (Lin et al., 2004). Interestingly, our results are in complete accordance with a study conducted by Morrison and Morrison where C57BL/6 mice genitally infected with *C. muridarum* demonstrated similar pattern (Morrison and Morrison, 2005).

In this study, we have developed the first *W. chondrophila* genital infection mouse model. Our results demonstrated that *W. chondrophila* genital infection resulted in organ pathology and a systemic bacterial burden that spread to cervix/vagina, lumbar lymph nodes, and spleen. The humoral immune response was IgG-mediated containing mainly IgG2a subtypes, thus suggesting Th1-skewed immunity.

## Author contributions

SV, JG, GG, and DB conceived and designed the experiments. SV, JG, LP, and MS performed the experiments. AP performed all the pathological interpretations. SV, JG, MS, and DB analyzed the data. SV and MS wrote the paper.

## Funding

This work was supported by the Department of Obstetrics and Gynecology, Maternity, Lausanne, Switzerland and by the SNSF grant number 310030_156169/1 attributed to DB. DB is also supported by the “Fondation Leenaards” through the “Bourse pour la relève académique,” by the “Fondation Divesa,” and by the “Loterie Romande.”

### Conflict of interest statement

The authors declare that the research was conducted in the absence of any commercial or financial relationships that could be construed as a potential conflict of interest.

## Supplementary material

Supplementary Figure 1 **Procedure of uterine horn infection and schematic timeline of experiments. (A)** Direct visualization of semi-rigid cannula in the uterine horn using ultrasound control (Voluson E8 General Electric Ultrasound probe 12 Mhz), which allows direct visualization of uterine infection (arrow). Heart is labeled H and spine is S. **(B)** Demonstration of uterine horn infection by semi-rigid cannula using blue dye. Regardless of which uterine horn the cannula is inserted in both uterine horns receive the blue dye indicating both left and right uterine horns and lumen would be infected using this procedure. **(C)** Schematic timeline of subcutaneous hormone injection, infection, sacrifice, vaginal wash, and bleeding of mice. Sacrifice included blood collection, vaginal wash, and organ harvesting.

Supplementary Figure 2 **Images of enlarged organs after genital ***W. chondrophila*** challenge exhibiting lymphadenopathy, splenomegaly, hepatomegaly, and severe metritis. (A, C, E)** organs from naive, uninfected mice. **(B, D, F)** enlarged organs (arrows) from mice infected with *W. chondrophila*.

Supplementary Table 1 **Sera MIF IgG from mice genitally infected with ***W. chondrophila*****.

Supplementary Table 2 **Percentage of mice exhibiting splenomegaly, lymphadenopathy, hepatomegaly, severe salpingitis and splenic, hepatic, and uterine horn leukocyte infiltration after ***W. chondrophila*** genital challenge**.
