# Supplementary material for: Waddlia chondrophila induces systemic infection, organ pathology, and elicits Th1-associated humoral immunity in a murine model of genital infection
Source: Front Cell Infect Microbiol. 2015 Nov 4;5:76. doi: 10.3389/fcimb.2015.00076 (PMC4631992; doi:10.3389/fcimb.2015.00076)
Supplement: Supplementary Figure 1 — Procedure of uterine horn infection and schematic timeline of experiments. (A) Direct visualization of semi-rigid cannula in the uterine horn using ultrasound control (Voluson E8 General Electric Ultrasound probe 12 Mhz), which allows direct visualization of uterine infection (arrow). Heart is labeled H and spine is S. (B) Demonstration of uterine horn infection by semi-rigid cannula using blue dye. Regardless of which uterine horn the cannula is inserted in both uterine horns receive the blue dye indicating both left and right uterine horns and lumen would be infected using this procedure. (C) Schematic timeline of subcutaneous hormone injection, infection, sacrifice, vaginal wash, and bleeding of mice. Sacrifice included blood collection, vaginal wash, and organ harvesting. [file DataSheet1.PDF]

Supplementary figure 1

A.

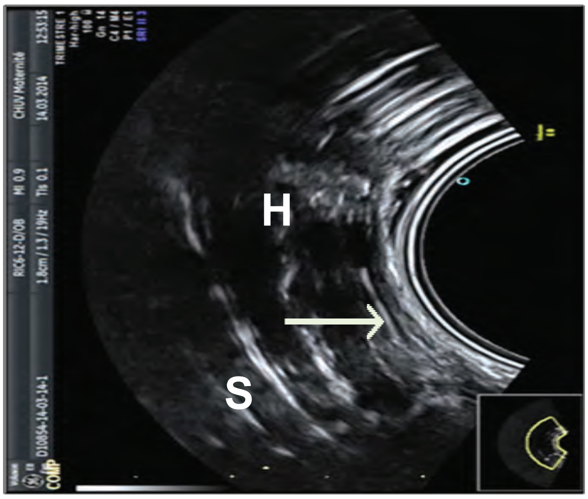

B.

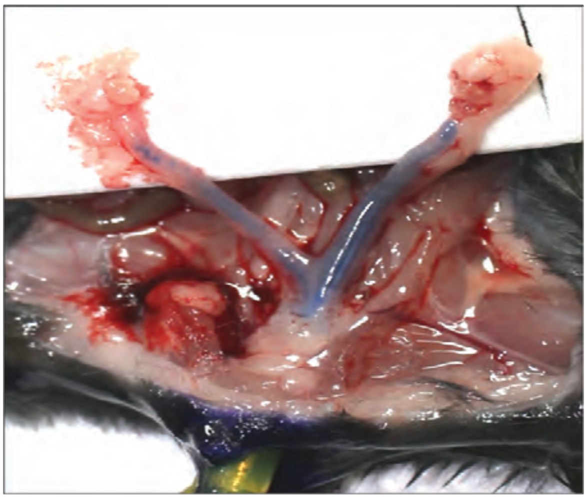

C.

Days	-9	-8	0	2	4	5	6	7	14	21	28	35	42	57
Estradiol	X													
Progesterone		X												
Waddlia infection			X											
Vaginal wash			X	X	X	X	X	X	X	X	X	X	X	
Bleed			X					X	X	X	X	X	X	X
Sacrifice				X				X	X	X				

Supplementary figure 2

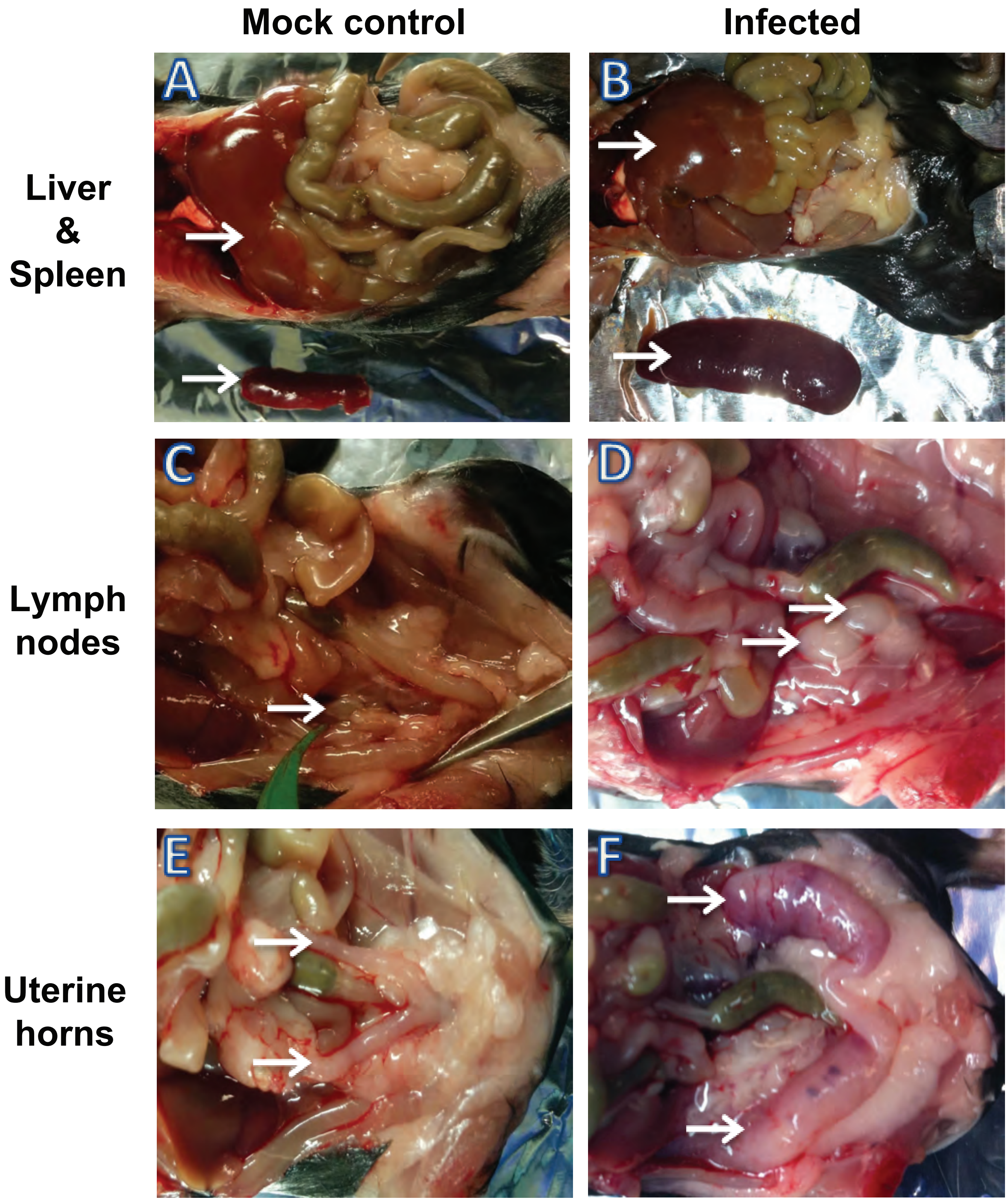

Supplementary table 1. Sera MIF IgG from mice genitally infected with *W. chondrophila*.

Days p.i	0	7	14	21	28	35	42	57
**Waddlia**	<1:32	<1:32	1:128	1:256	1:256	1:256	1:256	1:128
	<1:32	<1:32	1:128	1:256	1:256	1:128	1:128	1:128
	<1:32	<1:32	1:128	1:128	1:128	1:128	1:256	1:128
	<1:32	<1:32	1:64	1:128	1:128	1:128	1:128	1:128
	<1:32	<1:32	1:256	1:128	1:256	1:128	1:256	1:128
	<1:32	<1:32	1:256	1:128	1:256	1:256	1:256	1:256
	<1:32	<1:32	1:64	1:128	1:256	1:128	1:128	1:128
	<1:32	<1:32	1:256	1:256	1:256	1:512	1:256	1:256
	<1:32	<1:32	1:128	1:512	1:512	1:512	1:128	1:256
	<1:32	<1:32	1:128	1:128	1:512	1:256	1:256	1:256
	<1:32	<1:32	1:128	1:256	1:256	1:256	1:128	1:256
	<1:32	<1:32	1:128	1:512	1:256	1:256	1:128	1:256
**Mock**	<1:32	<1:32	<1:32	<1:32	<1:32	<1:32	<1:32	<1:32
	<1:32	<1:32	<1:32	<1:32	<1:32	<1:32	<1:32	<1:32
	<1:32	<1:32	<1:32	<1:32	<1:32	<1:32	<1:32	<1:32
	<1:32	<1:32	<1:32	<1:32	<1:32	<1:32	<1:32	<1:32
	<1:32	<1:32	<1:32	<1:32	<1:32	<1:32	<1:32	<1:32
	<1:32	<1:32	<1:32	<1:32	<1:32	<1:32	<1:32	<1:32
	<1:32	<1:32	<1:32	<1:32	<1:32	<1:32	<1:32	<1:32
	<1:32	<1:32	<1:32	<1:32	<1:32	<1:32	<1:32	<1:32
	<1:32	<1:32	<1:32	<1:32	<1:32	<1:32	<1:32	<1:32
	<1:32	<1:32	<1:32	<1:32	<1:32	<1:32	<1:32	<1:32
	<1:32	<1:32	<1:32	<1:32	<1:32	<1:32	<1:32	<1:32
	<1:32	<1:32	<1:32	<1:32	<1:32	<1:32	<1:32	<1:32

Supplementary table 2. Percentage of mice exhibiting splenomegaly, lymphadenopathy, hepatomegaly, severe salpingitis and splenic, hepatic and uterine horn leukocyte infiltration after *W. chondrophila* genital challenge.

	**Infected**			**Mock**		
Days p.i.	7	14	21	7	14	21
**Mice**						
Splenomegaly	25	**50**	25	0	0	0
Lymphadenopathy	50	**87**	37	0	0	0
Hepatomegaly	25	**50**	25	0	0	0
Severe salpingitis	12	**12**	25	0	0	0
**Spleen**						
Neutrophils	0	25	25	0	0	0
Macrophages	50	100	0	50	25	0
Lymphocytes	25	0	0	0	0	0
Necrosis	50	25	0	0	0	0
EMH	100	100	100	25	25	25
**Liver**						
Neutrophils	75	**100**	25	50	50	25
Macrophages	75	50	75	0	0	0
Lymphocytes	50	0	0	0	0	0
Necrosis	**100**	**100**	75	50	50	25
EMH	25	50	25	0	0	0
**Uterine Horn**						
Neutrophils	7	14	21	0	0	0
Macrophages	50	62	37	0	0	0
Lymphocytes	25	25	50	0	0	0
Necrosis	0	0	0	0	0	0
EMH	0	0	0	0	0	0
